# Simulation of Molecular Data under Diverse Evolutionary Scenarios

**DOI:** 10.1371/journal.pcbi.1002495

**Published:** 2012-05-31

**Authors:** Miguel Arenas

**Affiliations:** 1 Computational and Molecular Population Genetics Lab (CMPG), Institute of Ecology and Evolution, University of Bern, Bern, Switzerland; 2 Swiss Institute of Bioinformatics, Lausanne, Switzerland; Whitehead Institute, United States of America

This is an original *PLoS Computational Biology* tutorial.

## Introduction

This study is intended for evolutionary biologists interested in strategies for the simulation of molecular data under diverse evolutionary scenarios. It begins with a brief background on simulation approaches and describes some of the most important simulators developed to date. Then, several practical examples for simulating particular scenarios are presented, and finally, details are given on a variety of relevant applications of simulated data. Overall, this study provides a practical guide for applying simulation techniques to real world problems in molecular evolution.

## The Importance of Computer Simulations in Molecular Evolution

A commonly used methodology to mimic the processes that occur in the real world is to perform computer simulations [1]. Computer simulations allow us to understand which patterns may dramatically alter a particular system and can be used to study complex processes, including those that are analytically intractable. Furthermore, the simulation of multiple replicates with stochasticity may provide the variability required to study numerous processes, such as those often found in evolution. In molecular evolution, the simulation of genetic data has been commonly used for hypothesis testing (e.g., [2]), to compare and verify analytical methods or tools (e.g., [3]–[5]), to analyze interactions among evolutionary processes (e.g., [6]), and even to estimate evolutionary parameters (e.g., [7]). Consequently, a wide variety of tools have been developed to simulate sequence data under different substitution models of evolution, but also under different evolutionary processes such as selection, recombination, demographics, population structure, and migration. In recent years, new programs have been developed to handle very complex scenarios (e.g., [8], [9]) and efficient algorithms have been incorporated in order to accommodate large datasets in response to the increasing amount of genome-wide data (e.g., [10]). Thus, the importance of simulations continues to grow in order to deal with these new challenges.

## Approaches for the Simulation of Molecular Data

**Figure 1:**
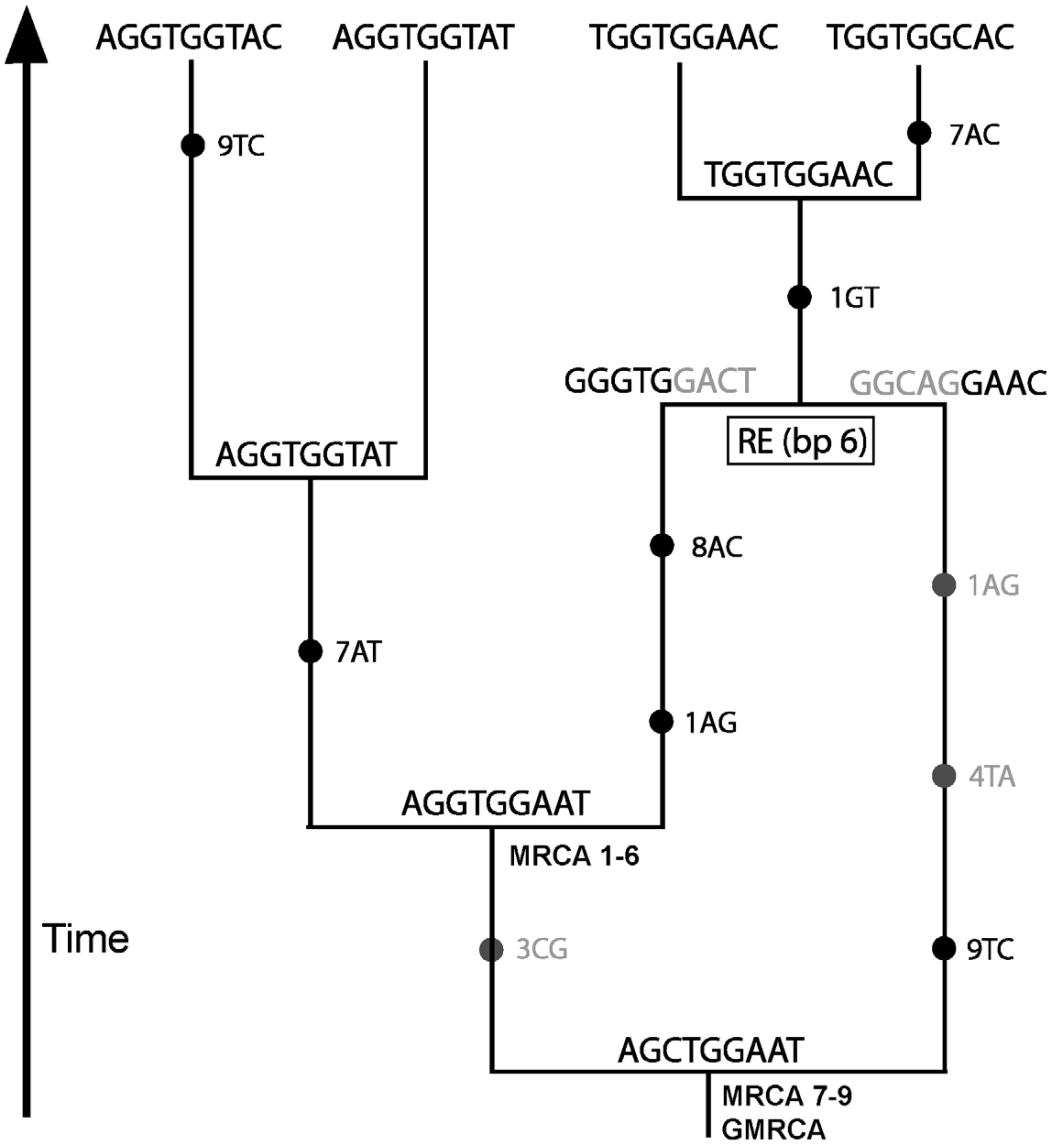
Example of nucleotide evolution on the ancestral recombination graph. Note that this ARG contains a recombination event with breakpoint at position 6. Starting from a sequence assigned to the GMRCA, substitutions (marked with black circles) occur forward in time. Non-ancestral material (material that does not reach the sample) and its substitution events are shown in grey.

After the simulation of evolutionary histories (see Box 1), or when just a rooted tree or network is given, a sequence assigned to the most recent common ancestor (MRCA, or grand MRCA [GMRCA] in the case of networks) can be evolved along branches according to a substitution model of evolution, in order to simulate sequences for all internal and terminal nodes (see an example in Figure 1). A common procedure consists of applying continuous-time Markov models defined by 4×4, 20×20, and 61×61 matrices of substitution rates for nucleotide, amino acid, and codon (note that stop codons are ignored) data, respectively (details in [11]). This methodology is very flexible and allows for heterogeneous evolution where different sites and branches can be evolved under different substitution models (e.g., [12]). These aspects suggest in practice two important considerations. Firstly, simulations of nucleotide sequences are much faster than simulations of coding or amino acid sequences due to the dimension of the substitution matrices. Secondly, a large number of branches (derived from a large number of taxa or recombination events) leads to slower simulations due to the need to re-calculate the matrix for each branch.

**Box 1. Simulation of Evolutionary Histories**

There are two main approaches commonly used to simulate evolutionary histories in population genetics: the forward in time (forward-time) and the coalescent (backward-time). Here I describe the main particularities of these approaches, considering goals and limitations for the simulation of diverse evolutionary scenarios.

The forward-time approach simulates the evolutionary history of an entire population from the past to the present and allows the success of a lineage to be a function of the genotype (see reviews, [13], [14], [80]). Thus, these simulations consider all ancestral information and therefore can be useful to fully study the subsequent evolutionary process of the population, including gene–gene interactions, mating systems, complex migration models (such as sex biased dispersal or long-distance dispersal), or complex selection (e.g., [42], [81], [82]); beginners may explore these basic concepts using educational simulations [83], [84]. Unfortunately, because the whole population history is simulated, forward simulations require generally extensive computational cost, although recently significant improvements have been achieved in this concern (e.g., [85]).

On the other hand, the coalescent approach describes a backwards in time genealogical process of a sample of genes to a single ancestral copy (see reviews [86], [87]). The coalescent allows the simulation of a limited set of scenarios, namely population size changes (e.g., [88]), population structure and migration (e.g., [89]), recombination (e.g., [90]), and selection (e.g., [91]). A key aspect of the coalescent is that the history of the whole population is not required (so it is not actually simulated) and, consequently, it is generally computationally faster than the forward-time approach. It is important to remember, however, that the efficiency of forward-time simulations is irrespective of the amount of recombination or selection, in contrast to coalescent simulations that are highly sensitive to such processes.

Coalescent and forward-time approaches can be considered complementary [13]. In fact, recently two new methods have incorporated both approaches for fast simulations of complex scenarios [9], [33]. In conclusion, one should keep in mind that the choice of the simulation approach may depend on the complexity of the target scenario, as well as on the required computational cost for the simulation.

## Main Software Implementations

A number of programs have been developed to simulate nucleotide, codon, and amino acid sequences evolution. Although several studies have already reviewed these software tools (e.g., [13]–[17]), such revisions quickly become obsolete due to the emergence of new simulators, as noted in [14]. Table 1 shows an updated list of user-friendly and commonly used programs available to date. Next, the most interesting software from a practical perspective is briefly described.

**Table 1:** The main software used to simulate genetic sequences under nucleotide, codon, and amino acid substitution models.

Program	Class	Process	Substitution Model	Rate Variation	Indels	OS	Ref.
SIMCOAL2	Coalescent	D, Pm, R	Nt: JC, K2P	No	No	Linux, Win	[65]
Fastsimcoal	Coalescent	D, Pm, R	Nt: JC, K2P	No	No	Linux, Mac, Win	[10]
Serial Simcoal	Coalescent	D, Pm	Nt: JC, K2P	No	No	SC, Mac, Win	[66]
mlcoalsim	Coalescent	D, Pm, R	Nt: JC, K2P	G, I	No	All	[67]
TREEEVOLVE	Coalescent	D, Pm, R	Nt: All	G	No	SC, Mac	[68]
CodonRecSim	Coalescent	R	Cod^c^: GY94	No	No	SC, Win	[23]
Recodon/NetRecodon^a^^,^ b	Coalescent	D, Pm, R	Nt: All; Cod^c^: GY94	G, I	No	All	[8], [24]
SPLATCHE2	Forward, Coalescent	D, Pm, R	Nt: JC, K2P	No	No	Linux, Win	[9]
AQUASPLATCHE	Forward, Coalescent	D, Pm	Nt: JC, K2P	No	No	Linux, Win	[20]
GenomePop	Forward	D, Pm, R^a^, S	Nt: JC, GTR; Cod: MG94	No	No	SC, Linux, Win	[18]
SFS_CODE	Forward	D, Pm, R, S	Nt: All; Cod: Nt^d^	G	Yes	All	[19]
SimuPop	Forward	D, Pm, R, S	Nt: All	No	Yes	All	[21]
EvolSimulator	Birth-death process^e^	D, Pm, S	Nt: All; Cod: Nt^d^; Aa: user defined	User defined^k^	No	SC	[69]
INDELible	Phylogenetic	-	Nt: All; Cod: GY94^f^, EM; Aa: 15 EM^g^	G, I	Yes	All	[12]
EVOLVER	Phylogenetic	-	Nt: All; Cod: GY94; Aa: 14 EM^h^	G, I	No	All	[28]
indel-Seq-Gen vs 2.0	Phylogenetic	-	Nt: All; Cod: Nt^d^; Aa: 6 EM	G, I	Yes	All	[27]
Seq-Gen	Phylogenetic	-	Nt: All; Cod: Nt^d^, Aa: 6 EM^i^	G, I	No	All	[26]
EvolveAGene 3	Phylogenetic	-	Cod: *E. coli* spectra	No	Yes	All	[70]
DAWG	Phylogenetic	-	Nt: All	G, I	Yes	All	[71]
MySSP	Phylogenetic	-	Nt: All	G	Yes	Win	[72]
SISSI	Phylogenetic	-	Nt: All; Cod: Nt^d^^,^ j	User defined^k^	No	All	[73]
ROSE	Phylogenetic	-	Nt: All; Aa: PAM	G	Yes	SC	[74]
SIMGRAM/SIMGENOME/GSIMULATOR	Phylogenetic	-	Nt: All; Cod: EM; Aa: Secondary structure	No	Yes	SC	[75]
ALF	Phylogenetic	-	Nt: F84, HKY, TN93, GTR; Cod: GY94 and EM; Aa: 6 EM^l^	G, I	Yes	All	[76]
SIMPROT	Phylogenetic	-	Aa: PAM, JTT, PMB	G	Yes	Linux, Win	[77]
PhyloSim	Phylogenetic	-	Nt: All; Cod: GY94^f^, EM; Aa: 9 EM^m^	G, I	Yes	R	[29]

“Class” includes phylogenetic (where a genealogy is already given from the user), forward, birth-death, and coalescent approaches. “Process” shows the implemented evolutionary scenarios: “D”, “Pm”, “R”, and “S” indicate demographics, population structure and migration, recombination, and selection, respectively. “Substitution model” refers to substitution models based on nucleotide “Nt”, codon “Cod”, and amino acid “Aa” sequences; indeed, “Nt: All” indicates all nucleotide substitution models developed so far (JC, …, GTR) and “EM” indicates empirical model. “Rate variation” indicates whether different sites can be evolved under different rates (G: gamma distribution; I: proportion of invariable sites). “Indels” indicates the consideration of insertion and deletion events. “OS” shows the availability of executable files and/or source code “SC” for different operative systems (“All” means that Macintosh, Windows, and Linux executables are available), and “R” means the R language for statistical computing. “Ref” indicates the reference of publication. Although many more software packages exist, here I have selected, from my point of view, those programs most commonly used, most user-friendly, and which implement the most diverse range of evolutionary scenarios.

a Intracodon recombination is also allowed in *NetRecodon* and *GenomePop*.

b The ARG can be exported from *NetRecodon* and can be then visualized and analyzed using *NetTest* [78].

c Under codon models, *ω* can change across codons.

d Coding sequences are simulated by nucleotide substitution models, avoiding stop codons.

e EvolSimulator simulates phylogenetic histories under the birth-death model of speciation and extinction [79].

f Under codon models, *ω* can change across codons and branches.

g Amino acid models implemented in *INDELible*: BLOSUM62, CpREV, DAYHOFF, DAYHOFF (DCMUT), HIVb, HIVw, JTT, JTT (DCMUT), LG, mtArt, MTMAM, mtREV, RtREV, VT, and WAG.

h Amino acid models implemented in *EVOLVER*: CpREV, CpREV64, DAYHOFF (DCMUT), DAYHOFF, GRANTHAM, JTT (DCMUT), JTT, LG, miyata, mtArt, MTMAM, mtREV24, mtZoa, WAG.

i Amino acid models implemented in *Seq-Gen*: BLOSUM62, CpREV24, JTT, mtREV, PAM, and WAG.

j Simulation of codons with structural dependency among sites.

k The rate of variation among sites can be introduced from the user.

l Amino acid models implemented in *ALF*: PAM, JTT, WAG, LG, CustomP, GCB.

m Amino acid models implemented in *PhyloSim*: CpREV, JTT, JTT (DCMUT), LG, mtArt, mtMam, mtREV24, mtZoa, WAG.

When attempting to simulate a complex evolutionary scenario, several programs developed under the forward-time approach may be useful (see Table 1). *GenomePop* [18] and *SFS_CODE* [19] seem the most comprehensive tools with implementations of population structure, demographic particularities, recombination, and selection, but they do not allow simulations under amino acid substitution models. The programs *SPLATCHE2* [9] and *AQUASPLATCHE* [20] are able to simulate nucleotide data under spatially (using land or freshwater maps, respectively) and temporally explicit demographic models. A disadvantage of these programs is that only two DNA substitution models are available, note that other programs such as *SFS_CODE* or *SimuPop* [21] implement all DNA substitution models (see Table 1), which may be problematic when trying to mimic genome-wide data (see [22]).

If our target scenario can be represented by the coalescent, a variety of coalescent-based programs are able to simulate nucleotide data (see Table 1). Nevertheless, only *CodonRecSim* [23], *Recodon* [24], and *NetRecodon* [8] can simulate coding sequences in the presence of recombination. The first two of these programs force recombination breakpoints to occur between codons while *NetRecodon* does not (see [8]). On the other hand, *fastsimcoal* [10], *Recodon*, and *NetRecodon* allow simulations with sampling at different times, which can be very interesting for the joint analysis of ancient and modern DNA [25].

When a phylogenetic history (one or several trees) is given, numerous programs exist to directly simulate sequences along such history (see Table 1, phylogenetic class). One of the most applied programs is *Seq-Gen* [26], which implements several nucleotide and amino acid substitution models. The program *indel-Seq-Gen 2.0* [27] extended *Seq-Gen* to include diverse indel (insertion and deletion) models. Almost at the same time as *Seq-Gen*, the program *EVOLVER* (from the *PAML* package [28]) was released, which additionally allowed the simulation of coding data. Recently, *INDELible* [12] and *PhyloSim* [29] implemented all those capabilities, and in addition they included codon models where dN/dS (nonsynonymous/synonymous rate ratio) may vary across sites and/or branches. *INDELible* is very user-friendly but *PhyloSim* was implemented in R (language for statistical computing, [30]) and requires some programming knowledge.

## Practical Examples

In this section I outline five hypothetical practical examples, of the fast simulation of genetic sequences under particular evolutionary scenarios, which will be of general interest. The reader may notice that some scenarios can be solved using more than one approach, but I base my suggestions here on how appropriate, flexible, and user-friendly I think the simulators are.

### I) Nucleotide Data under Natural Selection

This scenario is commonly applied to identifying targets of positive selection in real datasets (e.g., [31], [32]). To my knowledge, there is no coalescent framework available to simulate data under natural selection whilst using Markov DNA substitution models, which may bring realistic information because not necessarily every mutation occurs at a different site in the sequence. However, two programs can be combined to quickly perform this task. First, we can simulate coalescent trees using the programs *msms* [33] or *SelSim* [34], although both tools are limited to simulation of a single locus under selection. Then, nucleotide sequences can be evolved along those trees using *Seq-Gen*. Another possibility is to apply a forward-time simulator that implements complex selection and all DNA substitution models (e.g., *SFS_CODE*).

### II) Coding Data with Intracodon Recombination

Simulations with recombination breakpoints that occur within codons are more realistic since these particular events occur 2/3 of the time that a recombination happens, assuming a spatially uniform distribution. Therefore, these events might exert undue influence on other parameter estimates since current analytical phylogenetic methods using codon models and recombination assume intercodon recombination. However, such effects have not been observed; in particular, dN/dS estimations were not altered (see [8]), so this should be studied further. The fastest procedure for the simulation of intracodon recombination is to directly apply the program *NetRecodon*. Alternatively, *GenomePop* can also perform this simulation under the forward approach. This scenario was applied in [35].

### III) Amino Acid Data with Indels and Under Recombination

This is a very specific scenario, but one that can also be very interesting for readers due to its complexity and the multiple possible options for its simulation. For instance, this scenario could be useful for testing phylogenetic tree reconstruction (or recombination detection) methods from amino acid datasets that evolved under recombination (e.g., [36]). As far as I know, there is no single tool available that can simulate this scenario. My suggestion is to first simulate coalescent trees (a tree for each recombinant fragment) by the program *ms*, and then amino acid sequences with indels can be evolved on the respective trees using *INDELible*.

### IV) Long Genomic DNA Regions under Recombination

The amount of genomic data available increases rapidly and as a consequence, plenty of genetic studies focusing on large genomic regions have appeared (e.g., [37]). As expected, such studies require robust and memory-efficient simulators [10], [38]. One of them is *fastsimcoal*, which allows for efficient simulations because it is based on a simplification of the standard coalescent with recombination (the sequential Markovian coalescent [SMC] algorithm [39]). Therefore, it seems to be an appropriate framework to simulate this scenario.

### V) Coding Data under a Spatial and Temporal Range Expansion

Spatial and temporal range expansions have occurred repeatedly in the history of most species and promote genetic consequences that are different than those produced by pure demographic expansions [40]. In addition, other spatiotemporal processes, such as range contractions and range shifts (usually produced during climate changes) or long-distance dispersal events, can also affect molecular diversity [41], [42]. Using *SPLATCHE2*, trees can be simulated under spatial and temporal range expansion in a straightforward manner. Then, coding data can be simulated over those trees by *INDELible*.

## Applications of Simulated Genetic Data

Computer simulation is a powerful tool in population genetics with a rich variety of applications. Here I show some interesting published applications.

### I. Hypothesis Testing

1. The effect of recombination on ancestral sequence reconstruction.
  Recently, Arenas and Posada [35] tested if recombination can affect ancestral sequence reconstruction (ASR). They simulated nucleotide, codon, and amino acid data with *NetRecodon* and they observed that recombination biases the reconstruction of ancestral sequences, regardless of the method or software used. This effect was shown as a consequence of incorrect phylogenetic tree reconstructions when recombination is ignored [43]. Note that this effect is crucial for numerous ASR-based studies (e.g., [44]).
2. The effect of recombination on selection tests.
  Tests for identifying selection (based on dN/dS) are frequently used in different species, including highly recombining viruses and bacteria (e.g., [45]). There is, however, an important pitfall of such tests in the presence of recombination. In the studies [8], [23] authors simulated coding data under several heterogeneous codon models [46] and different levels of recombination. Then, they applied likelihood ratio tests (LRTs) for model choice. Results showed a weak impact of recombination on the estimation of global dN/dS but a strong effect at the local level by inflating the number of positively selected sites. Simulations were carried out using *CodonRecSim* and *NetRecodon*.
3. Testing criteria for substitution model selection.
  A common step in phylogenetics consists of the statistical selection of a DNA substitution model that best fits the data [47], [48]. Currently, this model selection can be performed using several criteria, namely hierarchical and dynamic LRTs, Akaike and Bayesian information criterion (AIC and BIC, respectively), and the decision-theoretic approach (DT). Although AIC and BIC showed advantages over LRTs [47], the best criterion among all other options remained unclear. Recently, Luo et al. [49] addressed this point by extensive simulations of nucleotide data (using *PAML* [28] to simulate four tree topologies and *Seq-Gen* to evolve DNA sequences under a wide set of substitution models) and coding data (using *Recodon*). Then, by statistical analysis they concluded that BIC and DT approaches favor accurate model selection.

### II. Verification of Analytical Methods

1. Validation of a method for large phylogenetic tree reconstruction.
  One of the most well-established programs for phylogenetic tree reconstruction is *PHYML* [50]. As with most analytical tools, *PHYML* required thorough validation through computer simulations. In particular, 5,000 random phylogenies were simulated according to the standard speciation process (see [51]), and then DNA sequences were evolved on those phylogenies using *Seq-Gen*. The program showed a topological accuracy similar to that from other maximum likelihood programs, but it strongly reduced computing time.
2. Validation of a method for the detection of recombinant breakpoints.
  Recombination detection methods are fundamental for the analysis of genome dynamics, genetic mapping, and phylogenetic methods. As a result, a variety of methods for recombination detection exist (see [52]). One of them was recently developed by Westesson and Holmes [5] for the analysis of whole-genome alignments. For its validation, ancestral recombination graphs (ARGs) were simulated using *Recodon*, then marginal trees with identical topologies were excluded and DNA sequences were simulated on the remaining trees using *Seq-Gen*. The method accurately detected recombinant breakpoints even for genome-size datasets.

### III. Study of Complex Evolutionary Processes

**Figure 2:**
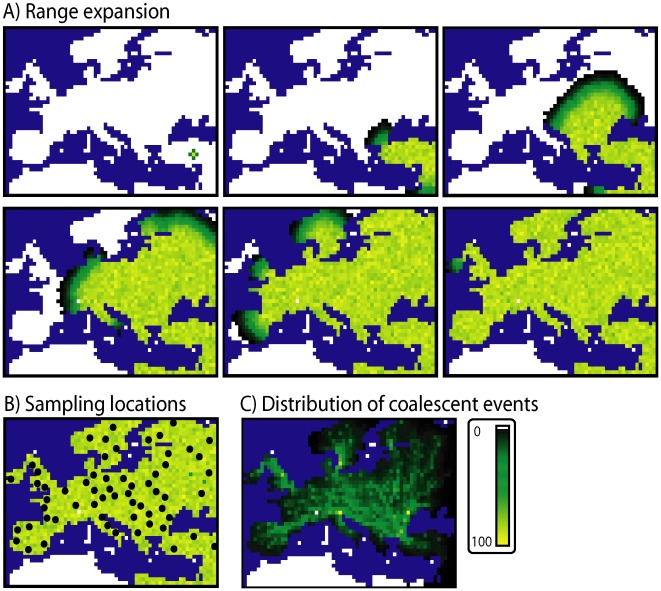
Example of a simulated modern human range expansion over Europe. (A) Snapshots of the program *SPLATCHE2* for an example of simulation of a Neolithic farmer expansion over Europe. Settings (demographic parameter values) used for this example are similar to those used in François et al. [55]. Note that the range expansion starts from the bottom-right corner of Europe. Snapshots are taken every 40 generations. White demes are empty and dark colors indicate low population densities (in particular at the front of the expansion). (B) Scheme of sampling locations used for this simulation. (C) Spatial distribution of coalescent events during the range expansion.

1. Principal component analysis of human genetic diversity across Europe.
  A controversial topic that sparked debate in recent years was the interpretation of gradients of population genetic variation across Europe derived from principal component analysis (PCA) [53]–[56]. Briefly, while initially Cavalli-Sforza et al. [56] interpreted principal component (PC) gradients only as a consequence of human ancestral expansions, Novembre and Stephens [53] showed that similar PC gradients may arise from diverse spatial genetic patterns under equilibrium isolation-by-distance models. Recently, François et al. [55] carried out simulations of DNA data using *SPLATCHE2* in order to mimic the Neolithic farmer expansion across Europe taking into account various levels of interbreeding between farmer and resident hunter-gatherer populations (see Figure 2). They concluded that demographic and spatial population expansions often lead to PC gradients that are perpendicular to the direction of the expansion as a consequence of the allele surfing phenomenon [57].

### IV. Estimation of Evolutionary Parameters

1. Coestimation of evolutionary parameters using approximate Bayesian computation.
  Approximate Bayesian computation (ABC) is a recent and useful approach for the inference in evolutionary genetics (see [58]), based on computer simulations. It provides a robust alternative for those analyses where the likelihood function cannot be evaluated or is computationally too expensive. An interesting example studied by Wilson et al. [59] applied ABC to coestimate several evolutionary parameters (such as mutation, dN/dS, and recombination rates) from coding data of the bacteria *Campylobacter jejuni*. Although the simulator used was not published, such a scenario could be simulated using e.g., *Recodon*. In addition, Laval et al. [60] also applied an ABC-based approach to coestimate, assuming a particular model of human evolution, important historical and demographic parameters like the onset of the African expansions and the out-of-Africa migration, as well as the current and ancestral effective population sizes of Africans and non-Africans. Here the simulation of DNA data was performed using *SIMCOAL2*.

## The Future of Computer Simulations

Although current software available can simulate a wide set of evolutionary scenarios, some limitations still remain concerning computational costs and particular complex models. In some cases the computational time is crucial (e.g., ABC studies that require millions of simulations to cover a wide range of parameter space), and running simulations in parallel on a cluster can help alleviate the computational time. On the other hand, several complex scenarios that interest evolutionary biologists are still difficult to simulate. An example is the simulation of molecular evolution with dependence among sites (coevolving sites, e.g., [61]). Here, although some models were already developed (see [62]), they could not be extensively applied in simulations due to intractable computational costs derived from the calculation of diverse structural energies (like those used in [63]). Another challenging scenario is the simulation of coding data under natural selection, but where the signatures of natural selection directly influence the synonymous and nonsynonymous substitutions (see [64]).

There is a permanent need of software for the simulation of molecular data due to the emergence of complex scenarios and the requirement of fast simulations. Thus, I expect a fruitful future for this basic and applied area of research.
